# Retrospective Long-Term Clinical Outcome of Feldspathic Ceramic Veneers

**DOI:** 10.3390/ma15062150

**Published:** 2022-03-15

**Authors:** Sorin Gheorghe Mihali, Dan Lolos, George Popa, Anca Tudor, Dana Cristina Bratu

**Affiliations:** 1 Department of Prosthodontics, Faculty of Dentistry, “Vasile Goldis” Western University of Arad, 94 Revolutiei Blvd., 310025 Arad, Romania; sorin@dentalconcept.org; 2 Department of Orthodontics and Dento-Facial Orthopedics, Orthodontic Research Center, Faculty of Dental Medicine, “Victor Babes” University of Medicine and Pharmacy, 2 Eftimie Murgu Square, 300041 Timisoara, Romania; bratu.cristina@umft.ro; 3 Medical Informatics and Biostatistics, Research Center in Dental Medicine Using Conventional and Alternative Technologies, Faculty of Dental Medicine, “Victor Babes” University of Medicine and Pharmacy, 9 Revolutiei 1989 Ave., 300070 Timisoara, Romania; atudor@umft.ro

**Keywords:** feldspathic ceramic, vertical prep, no-prep veneers, minimally invasive treatment

## Abstract

The purpose of this study was to evaluate the clinical outcome of feldspathic ceramic laminate veneers over a 7-year period using minimally invasive techniques, such as vertical preparation (without prosthetic finish line), or no preparation (no-prep). A total of 170 feldspathic ceramic veneers were cemented in the anterior region, including 70 maxillary and 100 mandibular veneers, after special conditioning of the teeth and restorations. The veneers were evaluated using the FDI World Dental Federation criteria evaluation kit after recalling all the patients between February and June 2021. In total, 14 feldspathic veneers failed and were replaced with lithium disilicate because of core fracture, and 10 cases of chipping occurred on the ceramic surface and were polished. The overall survival rate was 91.77% for up to 7 years of function, with a failure rate of 8.23%. In this retrospective survival analysis, the failures, including the fracture of veneers and dental hard tissue, occurred both in prep and no-prep teeth. No failures were observed in veneers with a maximum thickness of 0.5 mm compared to those with a maximum thickness of 1 mm, 1.5 mm, 2 mm, and 2.5 mm.

## 1. Introduction

All dental restorations are liable to failure; even in metal–ceramic prosthetic restorations, fractures can jeopardize function and esthetics [1].

Traditional metal–ceramic restorations have shown predictable strength, acceptable esthetic effects, and long-term survival in the oral environment [2]; however, a considerable amount of tooth structure must be removed during tooth preparation [3].

All-ceramic systems have been developed to achieve the most pleasing esthetic effect and expand treatment options for patients, causing a major shift in clinical workflow [4].

Abrasion and erosion are two increasingly common indications for dental treatment, and, if left untreated, may cause a loss of vertical dimension as well as diffuse and marked dentinal hypersensitivity [5,6]. To prevent these early-stage conditions, we must implement an appropriate treatment plan that preserves the dental hard tissue and ensures tooth vitality [7,8].

Owing to their strength, longevity, conservative nature, biocompatibility, and esthetics, feldspathic porcelain veneers are suitable prosthetic restorations in the frontal area, considering the long-term survival rates they achieve [9,10,11,12,13,14,15,16,17,18].

Several ceramic materials and fabrication methods are available for feldspathic porcelain veneers, including the platinum foil technique and the refractory die technique, which is the preferred technique in most laboratories [15,18]. Feldspathic ceramics also have desirable general properties such as flexural strength (62–90 MPa), compressive strength (172 MPa), shear strength (110 MPa), and modulus of elasticity (69 GPa) [19,20].

Several case reports present esthetic treatments that improve anterior tooth shape and alignment, size and proportions, and visibility of the teeth [21,22,23,24], re-establishing the esthetics and harmony of the patient’s smile using feldspathic ceramics.

Because of their high degree of translucency, feldspathic ceramics provide an excellent esthetic appearance that can optically approximate natural teeth [10,15,25]. Patients seeking esthetic treatment must undergo a comprehensive clinical examination including an esthetic evaluation [16,26], to ensure that the prosthetic restorations integrate into the relationship between the patient’s face and lip features.

In this retrospective study, we focused on the preparation of dental hard tissue by dividing patients into two groups: a vertical preparation group and a no-prep group. Owing to patient preferences, minimally invasive treatments currently provide the most impressive esthetic effect and excellent clinical performance with minimum thickness restorations [27,28,29,30,31], even in cases that require the removal of a small amount of dental hard tissue [7,10,16,24,28,32].

A vertical preparation [33,34] is a simple crown preparation (knife-edge, feather-edge, shoulder-less preparation) without a defined finish line, used to create a new anatomic crown with a prosthetic emergence profile [35]. We used this concept in a minimally invasive manner by removing just the surface of maximum convexity of the tooth structure. This vertical tooth preparation design was performed in all patients in the vertical preparation group.

Some clinical situations involve a minimal to no-preparation approach [5,10,24], or even step-by-step techniques [36,37,38,39], such as cases of severe dental erosion, which are treated with minimal, if any, tooth preparation.

Approaches such as additive-only as well as no-preparation [29,30,40,41,42,43] are excellent options for situations in which patients have healthy dental tissue.

In the case of dental hard-tissue loss, conventional restorations (crowns) normally require endodontic therapy and crown lengthening, procedures that are unable to efficiently conserve pulp vitality and meet the desired objectives [5,8,44]. In some situations, we used two separate feldspathic ceramics veneers with different paths of insertion to restore the affected anterior maxillary teeth. This approach considered certain parameters such as the emergence profile, the incisal translucency, and the positioning of the veneer margin in the point of maximum convexity of the teeth [40].

The combination of feldspathic ceramics and minimally invasive or noninvasive preparations provide clinicians with the option of a conservative approach in prosthodontic dentistry. In our study, we describe high-strength etchable ceramics that can be used at minimum thickness when the adhesive bonding methods of all-ceramic restorations are correctly used, as described by Ostermann [45].

However, the literature offers insufficient data regarding the survival rate and the clinical performance of feldspathic ceramic veneers over extended periods of function.

Accordingly, the aim of the present study was to evaluate the long-term clinical performance of feldspathic porcelain laminate veneers over a 7-year period, either using minimally invasive techniques such as vertical preparation without prosthetic finish line, or no preparation. The null hypothesis of the study was that there were no statistically significant differences between the clinical performance of feldspathic ceramic veneers with a maximum thickness of 0.5 mm, 1 mm, 1.5 mm, 2 mm, and 2.5 mm over a 7-year period.

## 2. Materials and Methods

### 2.1. Study Protocol

This retrospective study included 30 patients aged between 18 and 72 years, with a mean age of 36.7 ± 12 years. The patients (16 men and 14 women) visited our private dental clinic between 2013 and 2014; they were all treated by the same clinician (S.G.M.) and received 170 feldspathic ceramic veneers (70 applied on maxillary teeth and 100 on mandibular teeth). The study was approved by the Institutional Review Board of “Vasile Goldiș” Western University of Arad. All patients agreed to participate in the study and gave their written informed consent. Two methods were used: a minimally invasive/biologically oriented preparation (prep) technique for 108 veneers, and a no-preparation (no-prep) approach for 62 veneers. The veneers were also divided in five groups, according to their maximum thickness: 0.5 mm, 1 mm, 1.5 mm, 2 mm, and 2.5 mm. The biologically oriented preparation technique is a vertical preparation technique without a finish line, used to create a new anatomic crown with a prosthetic emergence profile. We opted for one of these two treatment options after carefully assessing several factors: tooth attrition, malocclusion, dental anomalies, and esthetic alteration.

Vital teeth without endodontic treatment or major crown destruction were eligible to be included in the study. Patients who presented any parafunctional signs and symptoms of bruxism according to the International Classification of Sleep Disorders [46] were excluded from veneer treatment at the time of arrival. Patients that had severely malpositioned teeth were first referred for orthodontic treatment to allow a minimally invasive approach that avoided sacrificing healthy tooth structure in excess.

All patients were monitored periodically for at least 2 years after they received the veneers.

**Figure 1:**
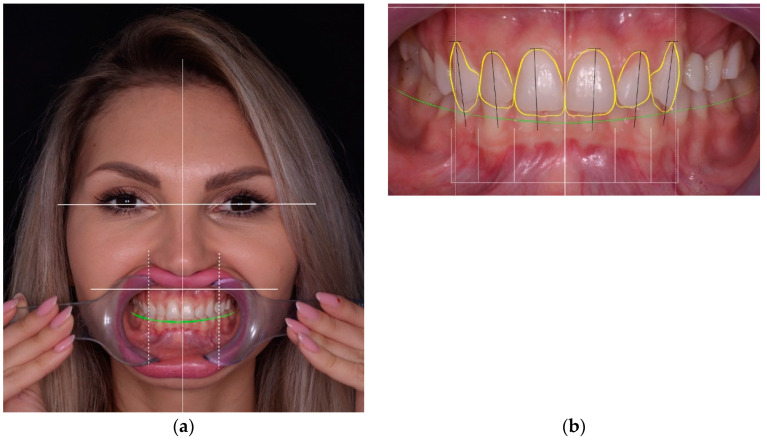
Esthetic and functional analysis of a patient: (**a**) facial analysis; (**b**) dento-gingival analysis.

**Figure 2:**
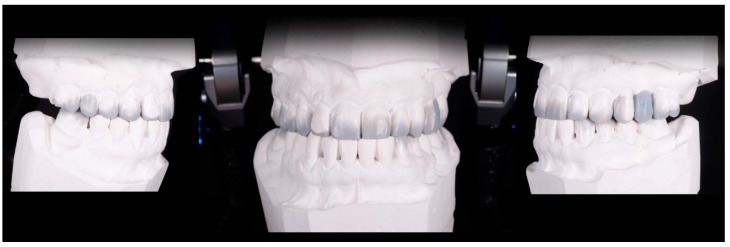
Diagnostic wax-up in which minimally invasive corrections with wax have been made. In all cases, the first option was an additive wax-up instead of substrative.

**Figure 3:**
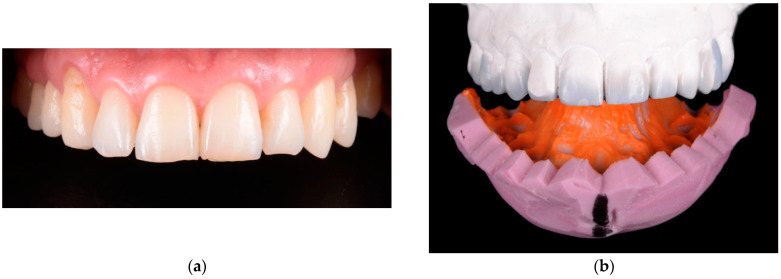

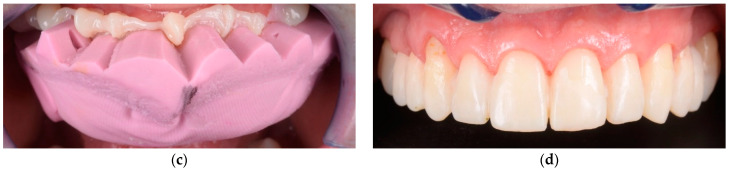
Direct mock-up: (**a**) initial situation; (**b**) silicon index made by diagnostic wax-up; (**c**) intraoral fabricated with a temporary resign material and silicon index; (**d**) clinical aspect of mock-up after finishing.

**Figure 4:**
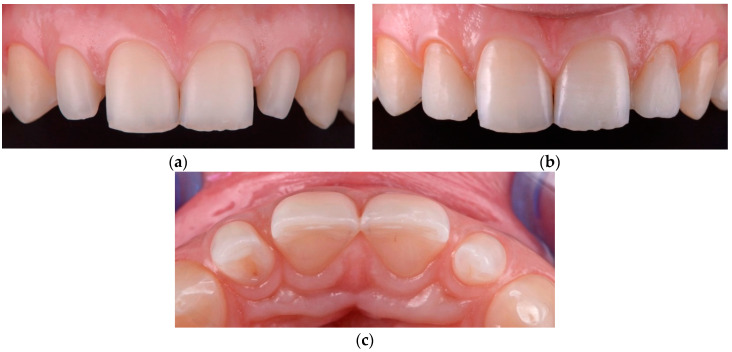
A microdontia of the lateral incisor: (**a**) initial situation; (**b**) additive direct mock-up, with enough space for final restoration; (**c**) no-prep technique applied by removing the composite from the natural teeth and preparing for impression.

**Figure 5:**
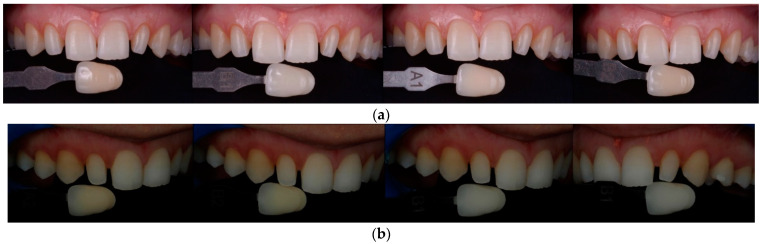
Color matching with: (**a**) VITA classical shade guide and (**b**) polar_eyes cross-polarization filter (Bio-Emulation™, Freiburg im Breisgau, Germany).

All restorations were made following a case set-up protocol. First, the patients underwent an esthetic and functional analysis (Figure 1), and impressions were taken. Subsequently, a diagnostic wax-up was made for the teeth that required treatment (Figure 2). Using a temporary resin material (Protemp 4, 3M ESPE, Seefeld, Germany) a direct mock-up was made by transferring the wax-up into the mouth of each patient (Figure 3). After 1–2 weeks of testing the provisionals, the decision regarding minimal or no preparation was made, based on the esthetic and functional preferences of each patient. In cases that presented enough space for the final restoration, with a minimal convexity of the teeth and a favorable color, the no-prep technique was applied (Figure 4). The final impression was made with polyvinylsiloxane (Virtual 380, Ivoclar Vivadent, Schaan, Liechtenstein) using a single-impression double-mixing technique with a standard tray after the mock-up was removed from the teeth (Figure 4c). The color was recorded (Figure 5) with a VITA classical shade guide and a polar_eyes cross-polarization filter (Bio-Emulation™, Freiburg im Breisgau, Germany) (Figure 5b).

**Figure 6:**
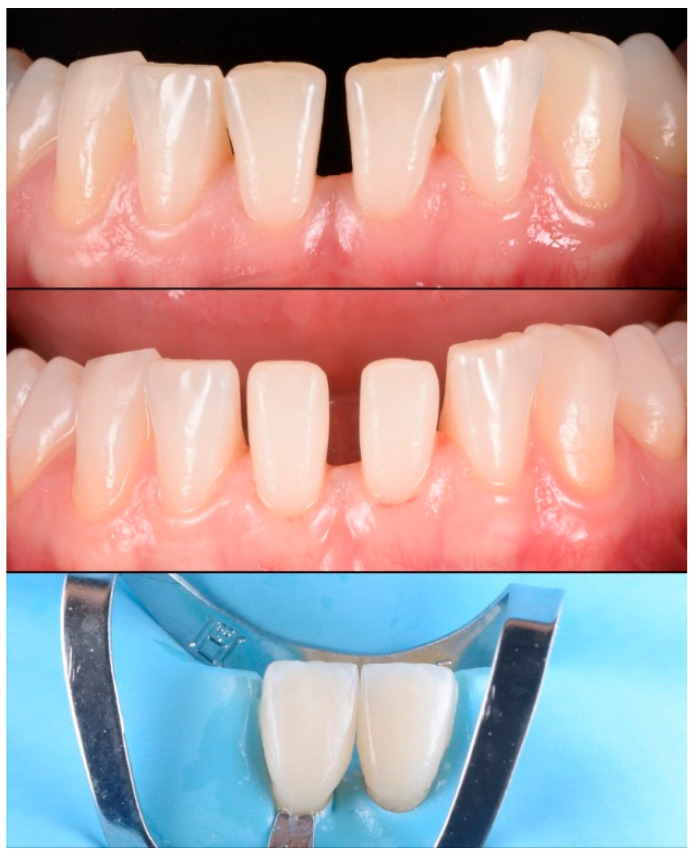
Minimally invasive preparation (vertical preparation) in central incisors for closing a diastema.

**Figure 7:**
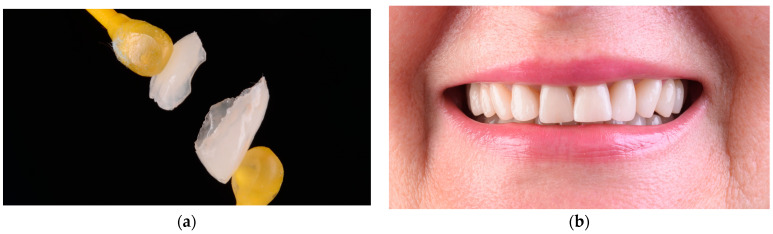
Noninvasive restorations without preparation of teeth: (**a**) a double veneer technique using feldspathic ceramic; (**b**) final clinical result with feldspathic double veneer ceramic restorations.

In the group in which we applied minimally invasive techniques, the objective was to obtain space for the future restoration, removing the maximum tooth convexity and any existing undercuts (Figure 6). The teeth were prepared using magnifying loupes (x4.3, Zeiss, Wetzlar, Germany) directly in the mock-up for reducing errors. As alternatives to full crowns, double veneers were used instead with a prepless technique (Figure 7).

Provisional restorations were prepared to maintain tooth position, function, and esthetics using a mock-up as described previously. Using a double-mixing technique, a full-arch, polyvinylsiloxane (ExpresXT, 3M ESPE, Seefeld, Germany) single impression was made for the opposing arch, and a type IV dental stone (GC Fujirock EP, Tokyo, Japan) was poured immediately.

**Figure 8:**
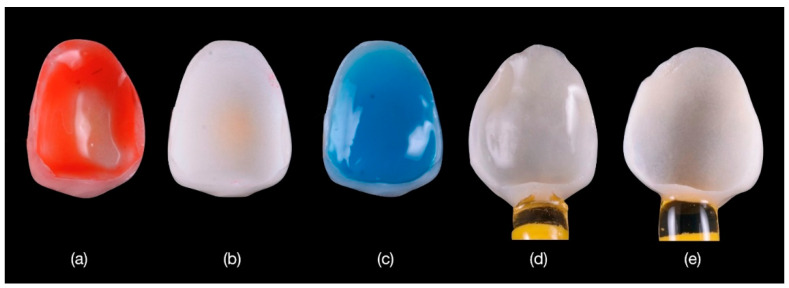
(**a**) Conditioning the feldspathic veneers with hydrofluoric acid applied for 60 s then (**b**) washed and rinsed with water and dried; (**c**) crystalline debris precipitate at the ceramic surface was removed with 36% orthophosphoric acid; (**d**) the etched surfaces were silanized with Monobond Plus for 60 s; and (**e**) dried to obtain a monolayer of silane.

**Figure 9:**
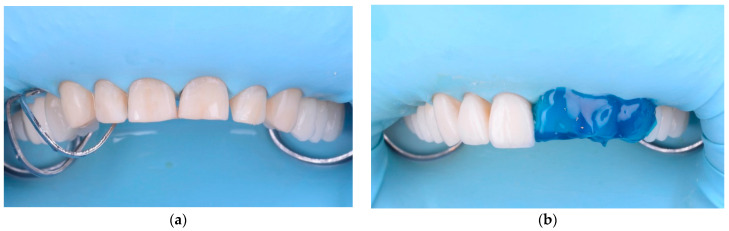

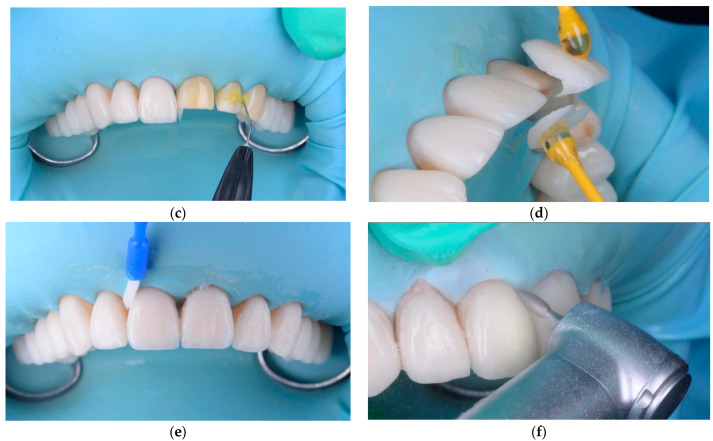
Intraoral cementation: (**a**) the rubber dam was applied; (**b**) the tooth was etched with 36% orthophosphoric acid for 45 s; (**c**) adhesive was applied and dried until a monolayer was obtained; (**d**) the veneers were cemented; (**e**) the excess cement was removed with a brush; (**f**) the margins were finished to obtain a good adaptation after cementation.

All restorations were made by the same dental technician following the manufacturer’s instructions. Each restoration was placed on the tooth after removing the provisional mock-up and assessed for proximal contacts, marginal adaptation, occlusal relationships, and shade matching. Veneers were then etched for 60 s with 3% to <7% hydrofluoric acid (IPS Ceramic Etching Gel, Ivoclar Vivadent, Schaan, Liechtenstein), washed and rinsed with water, and dried. Since etching with hydrofluoric acid leaves a significant amount of crystalline debris precipitate on the ceramic surface, the feldspathic restorations were also cleaned for 60 s using 36% orthophosphoric acid (Blue Etch, Cerkamed, Stalowa Wola, Poland) (Figure 8c) and ultrasonically cleaned in distilled water for 5 min. Thereafter, the etched surfaces were silanized (Monobond Plus, Ivoclar Vivadent, Schaan, Liechtenstein) for 60 s (Figure 8d) and dried to obtain a monolayer of silane (Figure 8e) [47]. A rubber dam was placed (Figure 9a), and the implicated teeth were sandblasted with 50 µm Al₂O₃ (Aluminium Oxide, RØNVIG Dental Mfg. A/S, Daugaard, Denmark). The enamel of the tooth was etched with 36% orthophosphoric acid (Figure 9b) for 45 s (Blue Etch, Cerkamed, Stalowa Wola, Poland), rinsed with water, and air dried. Finally, adhesive (Adhese Universal VivaPen, Ivoclar Vivadent, Schaan, Liechtenstein) was applied and dried until a monolayer was obtained (Figure 9c). A dual-cure luting resin cement was used (Variolink Esthetic LC, Ivoclar Vivadent, Schaan, Liechtenstein). Before polymerization, excess resin material was removed using a microbrush and hand instruments (Figure 9e). Both proximal sides of the restorations were light-cured for 30 s, with a light intensity of approximately 1.470 mW/cm^2^. A glycerin gel was applied on the restorations and the polymerization procedure was performed for an additional 20 s to eliminate the oxygen inhibition layer. Any excess cement was removed with a scaler, dental floss, and a double-edged 12D surgical blade. The accessible restoration margins were further finished (Figure 9f) and polished using the OptraFine Diamond Polishing System (Ivoclar Vivadent, Schaan, Liechtenstein) and interproximal polishing strips (Diamond Strips, Komet, Lemgo, Germany). The rubber dam was removed, and any final occlusal adjustments were made in centric relation, in lateral and protrusive movements. The ceramic surface was then polished with felt polishing wheels and polishing paste (OptraFine HP Polishing Paste, Ivoclar Vivadent, Schaan, Liechtenstein).

After cementation, periodic evaluations were performed every 6 to 12 months (during routine professional dental hygiene appointments) by two dentists who did not participate in the restorative procedure at the time of cementation (baseline). For the final re-evaluation, after at least 7 years of function, all the patients were recalled between February and June 2021.

The restorations were evaluated for apparent changes in their external structural integrity and marginal integrity. Secondary caries, fracture of the restoration/tooth, debonding of the indirect restoration, severe periodontal breakdown, or pulpal necrosis were considered absolute failures. A clinical qualitative evaluation was performed using the FDI World Dental Federation criteria [48,49] for assessing direct and indirect dental restorations considering three main indicators (a total of 16 items): biological (six items), functional (six items), and esthetic (four items). In our study, we were able to investigate 11 items (Table 1). Each veneer was clinically examined, and a score was given for each criterion (item) on a 5-point scale (e.g., 1, an excellent restoration; 5, a restoration that required replacement). Each examinator used an intraoral mirror, sharp explorer, and periodontal probe specifically designed for evaluating the FDI World Dental Federation criteria. All the veneers received a baseline score of 1 (excellent restoration) at the moment of cementation.

### 2.2. Statistical Analysis

Collected data were statistically analyzed using the SPSS Statistics platform (SPSS Inc. Released 2008. SPSS Statistics for Windows, Version 17.0. Chicago, IL, USA: SPSS Inc.). Nonparametric statistical tests (Mann–Whitney U, Kruskal–Wallis H, Wilcoxon Signed Ranks) were used to analyse the categorical data sets. The survival rate of the indirect restorations was calculated using the Log Rank (Mantel-Cox) test. The tests were considered statistically significant for *p* < 0.05. The feldspathic ceramic veneers were analyzed starting at baseline and ending when the clinician determined that an irreparable failure of the restoration occurred. When a veneer failed, it was replaced with a new lithium disilicate restoration.

## 3. Results

### 3.1. Comparison between the Prep and No-Prep Technique

No statistically significant differences were found between the maximum thickness (mm) of the veneers used in the no-prep and prep method (Mann–Whitney U Test, *p* = 0.814).

The Mann–Whitney U test results for comparing the prep and no-prep methods for each FDI criterion after a 7-year period showed that the scores for the no-prep method were significantly higher than the scores for the prep method for C3 (*p* = 0.038), C6 and C10 (*p* < 0.001), and for C8 (*p* = 0.001), respectively.

### 3.2. Comparison between the Five Groups of Maximum Veneer Thickness

The Kruskal–Wallis H test results for comparing the five groups of maximum veneer thickness (0.5 mm, 1 mm, 1.5 mm, 2 mm, and 2.5 mm) for each FDI World Dental Federation criterion after a 7-year period showed statistically significant differences for the following criteria: C1 (*p* < 0.001), C3 (*p* < 0.001), C4 (*p* < 0.001), C6 (*p* < 0.001), C7 (*p* = 0.004), C8 (*p* < 0.001), C9 (*p* = 0.001), and C10 (*p* < 0.001).

Using the Mann–Whitney U test, we further refined the results, comparing the maximum veneer thickness groups in pairs for each FDI criterion after a 7-year period (Table 2).

For the C1, C4, C6, C8, and C10 criteria the scores for the veneers with a maximum thickness of 0.5 mm were significantly lower than those for the veneers with a maximum thickness of 1 mm.

With the exception of the C9 criterion, the scores for the 0.5 mm group were significantly lower than those for the 1.5 mm group.

With the exception of the C7 criterion, the scores for the 0.5 mm group were significantly lower than those for the 2 mm group and the 2.5 mm group.

For the C7, C8, and C10 criteria the scores for the 1 mm group were significantly lower than those for the 1.5 mm group.

For the C3, C4, C8, C9, and C10 criteria the scores for the 1 mm group were significantly lower than those for the 2 mm group.

For the C4 criterion the scores for the 1 mm group were significantly lower than those for the 2.5 mm group.

For the C3, C6, and C10 criteria the scores for the 1.5 mm group were significantly lower than those for the 2 mm group.

No statistically significant differences were found for any score when comparing the 1.5 mm and 2.5 mm groups, and the 2 mm and 2.5 mm groups.

### 3.3. Comparison between the Baseline Scores and the Scores after 7 Years

The Wilcoxon Signed Ranks test results (*p*-values) after comparing the baseline scores (T1) and the scores after 7 years (T7) for each FDI criterion are presented in Table 3.

For the C1, C3, C4, C6, C7, C8, C9, and C10 criteria, the overall scores after 7 years are significantly higher than the baseline scores.

For the C1, C3, C4, C6, C8, and C10 criteria, the scores after 7 years for the no-prep method are significantly higher than the baseline scores.

For the C1, C3, C4, C6, C7, C8, C9, and C10 criteria, the scores after 7 years for the prep method are significantly higher than the baseline scores.

All the feldspathic ceramic veneers were rated clinically excellent for color match and translucency even after 7 years of function (score 1).

The functional properties on marginal adaptation were also very good (score 1).

Regarding the biological properties, postoperative sensitivity and tooth vitality were rated as excellent (score 1) for the no-prep method, while the adjacent mucosa was rated as excellent (score 1) for both methods. No secondary caries or pulpal necrosis was observed in the natural teeth.

### 3.4. Survival Time Analysis

Out of 170 veneers, 156 veneers (91.77%) survived after 7 years of function, with a failure rate of 8.23%. A total of 14 feldspathic veneers completely failed due to core fracture and were replaced with lithium disilicate restorations.

**Figure 10:**
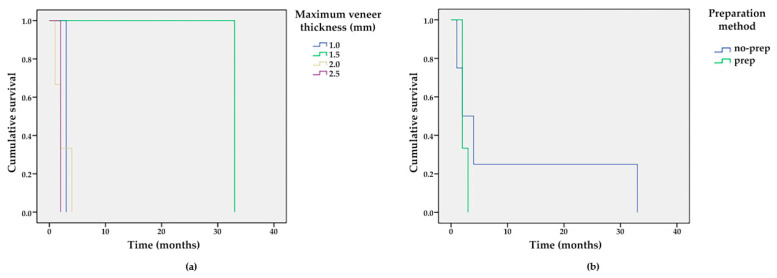
Survival functions for different (**a**) veneer thicknesses and (**b**) preparation methods.

The failures were both on prep and no-prep teeth, but no failures were observed for the veneers with a thickness of 0.5 mm (Figure 10 and Table 4). No statistically significant differences were found between the time-dependent survival curves both for the prep and no-prep methods (Log Rank (Mantel-Cox), *p* = 0.247), and for the five thickness groups (Log Rank (Mantel-Cox), *p* = 0.065).

**Figure 11:**
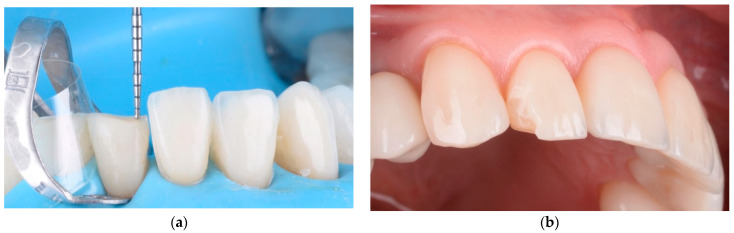
(**a**) When the thickness of the veneers exceeded 2 mm, future fractures could result because of unsustained ceramic; (**b**) chipping of the feldspathic ceramic veneers was observed.

**Figure 12:**
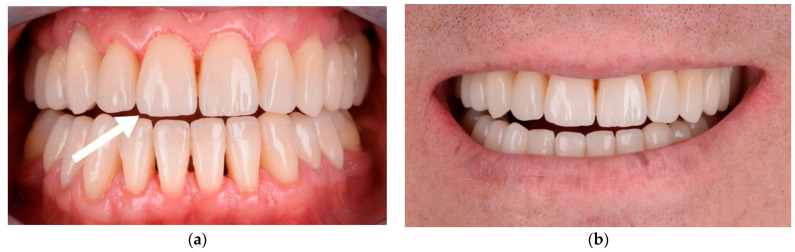
(**a**) Minor chipping on the central right upper incisor; (**b**) the chipping was managed by finishing and polishing.

We observed that the veneers with a thickness higher than 1.5–2 mm were more susceptible to fracture (Figure 11b); the fractures implicated both the veneers and dental hard tissue. Minimal chipping was observed on the ceramic surface of 10 veneers, in cases where the space for the ceramic was under 1 mm (Figure 12a). These surfaces were finished and polished without replacing the veneers (Figure 12b).

## 4. Discussion

In this retrospective study, we evaluated the clinical outcome of feldspathic veneers placed in our private practice for up to 7 years. Feldspathic veneers had a survival rate of 91.77%, which is comparable with the results of previous studies [12,35,50]. These studies report several clinical outcomes [12,35], with an overall estimated cumulative survival rate between 87–89% [12], while the most frequent complication was fracture/chipping. Compared to prior studies reporting high survival rates of feldspathic and porcelain veneers (1), our study considered secondary caries and endodontic complications absolute failures in terms of the long-term success of such restorations. Most of the failures were observed in the first 6 months, after cementation. Regarding the outcome of the vertical preparation group, similar results were observed in previous studies, in which the vertical preparation method was considered a promising alternative to horizontal preparation [50].

Regarding the esthetic objectives, we found similarities with other studies [35], which aimed to use a preparation method that could fix an anatomical crown with a prosthetic emergency profile and integrate it harmoniously with the teeth already present in the anterior region of the dental arches.

When using minimally invasive or no-prep techniques, the vertical dimension of occlusion (VDO) can be safely increased for up to 5 mm in the anterior region without causing pain or discomfort [51]; the patient can rapidly adapt to the new VDO within 2 weeks, if the temporomandibular joints are healthy and the articular disks are properly aligned. Utilizing feldspathic ceramic restorations in cases of dental attrition is an efficient way to increase the VDO.

The accuracy of the new VDO can be verified with the phonetic test by analyzing the pronunciation of words, particularly the use of the “s” consonant [52], as well as using provisional restorations [28], which aid both in restoring the physiognomic function and maintaining an optimal position for the final restorations. Direct mock-ups can also be used to test the modified VDO.

Although the materials used in all-ceramic restorations have been recommended for fabricating inlays/onlays [53,54,55] and single anterior and posterior crowns [56], these ceramic materials are not strong enough in the posterior region without adequate enamel support. One recent study [57] demonstrated that lithium disilicate overlays can increase the VDO using minimally invasive techniques, and show a 32-month survival rate of 97.7%. The average amount of dental tissue removed was less than 1 mm (0.98 mm in nonfunctional cusps, 0.88 mm in functional cusps, and 0.57 mm in the central fossa). The study concluded that lithium disilicate posterior overlays provided a complication-free treatment option, with an excellent survival rate, and the material allowed for conservative restorations with minimum thickness if the adhesive cementation technique was taken into consideration.

Generally, higher tensile and shear stress occurs when there are large areas of unsupported feldspathic ceramic (as in cases of diastema closure and teeth with chipping [12] or fracture), because these materials are too weak when the ceramic material must be extended more than 2 mm beyond the surface of the tooth [58]. We also observed that in spaces larger than 1.5 mm, the ceramic is susceptible to cracks (Figure 10b).

The final color of the tooth is affected by the thickness of the restoration, substrate color, ceramic color, and cement shade [59,60]. Feldspathic ceramic made on a refractory cast can restore tooth shape and color effectively [60]; however, it cannot be used to mask a darkened dental substrate [24,61], because these types of restorations are indicated in cases that require only a slight color change. In all cases of tooth discoloration or when a color change is desired, feldspathic ceramic restorations are not indicated. In our study, the restorations in both groups (the vertical preparation group and no-prep group) were performed by the same technician using the refractory die technique [18].

A photoshop assessment and functional evaluation, as well as a wax-up and mock-up, were used during pre-prosthetic planning. The esthetic properties of such restorations depend on the technician’s ability to properly replicate the anatomy, color, and translucency into the restoration; therefore, a strong collaboration between the patient, dentist, and technician is crucial [10,14].

An advantage to feldspathic ceramics is the absence of a core material, which allows increased space for characterization in the middle and incisal thirds; some of these materials showed excellent antibacterial performance owing to the 30% nanosized percentage of Ag ions [62]. With this technique, it is possible to minimize the tooth preparation and avoid over-contouring of the future restoration; the veneers can be aligned with the surface of the enamel, on the same level as the dental hard tissue [40]. After adhesive cementation, feldspathic ceramics allow for much easier finishing and polishing of the veneer margins than other ceramic materials.

The minimally invasive adhesive treatment is limited only to vital teeth without discoloration. The preservation of tooth structure and the remaining enamel should provide sufficient resistance strength, even in the presence of reduced thickness of the ceramic feldspathic material.

In a quality assessment of dental treatments using en-face optical coherence tomography, Sinescu et al. [63] found microleakage at prosthetic interfaces and material defects in several types of prosthetic restorations. Future studies should assess long-term veneers and their related complications, using optical coherence tomography (OCT) to analyse the marginal closure in more detail. Another technique that can be taken into consideration is the use of an intraoral scanner to make the refractory model. These digital impressions seem to be a viable alternative of analog technique [64].

## 5. Conclusions

The results obtained in this retrospective study showed that the use of feldspathic ceramic veneers in a private general practice, using minimally invasive preparation methods, achieved an overall success rate of 91.77% over a period of 7 years, on vital teeth without major crown destruction. The failures, including the fracture of the veneers and dental hard tissue, occurred both in prep and no-prep teeth. No failures were observed in the veneers with a maximum thickness of 0.5 mm, compared to those with a maximum thickness of 1 mm, 1.5 mm, 2 mm, and 2.5 mm. No statistically significant differences were found between the time-dependent survival curves for the two methods, and for the five thickness groups.

No secondary caries or pulpal necrosis were observed. The postoperative sensitivity and tooth vitality were rated as excellent for the no-prep method. All the feldspathic ceramic veneers were rated clinically excellent for color match, translucency, marginal adaptation, and adjacent mucosa, both for the prep and the no-prep method.

## Figures and Tables

**Table 1:** List of FDI World Dental Federation criteria used in our study to assess the esthetic, functional, and biological properties for the clinical evaluation of indirect restorations.

**Esthetic properties**		
C1 = Staining: (a) surface and (b) margin	C2 = Color match and translucency	C3 = Esthetic anatomical form
**Functional properties**		
C4 = Fracture of restorative material and retention	C5 = Marginal adaptation	C6 = Occlusal contour and wear
**Biological properties**		
C7 = Postoperative sensitivity and tooth vitality	C8 = Recurrent caries	C9 = Tooth integrity
C10 = Periodontal response (always compared to a reference tooth)	C11 = Adjacent mucosa	
**Score**		
1 = Clinically excellent/very good	2 = Clinically good	3 = Clinically sufficient/satisfactory
4 = Clinically unsatisfactory (but reparable)	5 = Clinically poor (replacement necessary)	

**Table 2:** Mann–Whitney U test results (*p*-values) after comparing the maximum veneer thickness groups in pairs for each FDI criterion after a 7-year period.

Maximum Thickness (mm)	0.5/1	0.5/1.5	0.5/2	0.5/2.5	1/1.5	1/2	1/2.5	1.5/2	1.5/2.5	2/2.5
	*p*-Value									
C1_T7	<0.001 *	0.001 *	0.001 *	0.007 *	0.532	0.302	0.903	0.119	0.601	0.765
C3_T7	0.052	0.025 *	0.025 *	0.001 *	0.679	<0.001 *	0.108	<0.001 *	0.151	0.849
C4_T7	0.012 *	0.005 *	0.005 *	<0.001 *	0.382	0.012 *	0.029 *	0.054	0.080	0.605
C6_T7	<0.001 *	0.001 *	0.001 *	0.017 *	0.676	0.080	0.765	0.048 *	0.917	0.238
C7_T7	1.000	0.010 *	1.000	1.000	0.016 *	1.000	1.000	0.145	0.643	1.000
C8_T7	0.007 *	<0.001 *	<0.001 *	<0.001 *	<0.001 *	<0.001 *	0.089	0.664	0.345	0.285
C9_T7	1.000	0.071	0.011 *	<0.001 *	0.094	0.018 *	0.270	0.467	0.410	0.605
C10_T7	0.007 *	<0.001 *	<0.001 *	<0.001 *	0.017 *	<0.001 *	0.089	0.021 *	0.482	0.531

C1–C10—each criterion is explained in Table 1; T7—the score after 7 years; * *p* < 0.05.

**Table 3:** Wilcoxon Signed Ranks test results (*p*-values) after comparing the baseline scores (T1) and the scores after 7 years (T7) for each FDI criterion.

	Overall Score	Score for N Method	Score for P Method
	*p*-Value		
C1_T7—C1_T1	<0.001 *	<0.001 *	<0.001 *
C2_T7—C2_T1	1.000	1.000	1.000
C3_T7—C3_T1	<0.001 *	<0.001 *	<0.001 *
C4_T7—C4_T1	<0.001 *	<0.001 *	<0.001 *
C5_T7—C5_T1	1.000	1.000	1.000
C6_T7—C6_T1	<0.001 *	<0.001 *	<0.001 *
C7_T7—C7_T1	0.046 *	1.000	0.046 *
C8_T7—C8_T1	<0.001 *	<0.001 *	<0.001 *
C9_T7—C9_T1	0.014 *	0.157	0.046 *
C10_T7—C10_T1	<0.001 *	<0.001 *	0.014 *
C11_T7—C11_T1	1.000	1.000	1.000

C1–C11—each criterion is explained in Table 1; T1—the baseline score; T7—the score after 7 years; N—no-prep; P—prep; * *p* < 0.05.

**Table 4:** Means and medians for survival time (in months), based on the preparation method and the maximum thickness of the veneers.

	Mean				Median			
Parameter	Estimate	Std. Error	95% Confidence Interval		Estimate	Std. Error	95% Confidence Interval	
			Lower Bound	Upper Bound			Lower Bound	Upper Bound
**Preparation method**								
N	10.000	5.036	0.130	19.870	2.000	1.061	0.000	4.079
P	2.333	0.211	1.920	2.747	2.000	-	-	-
Overall	6.714	2.986	0.862	12.567	2.000	0.309	1.395	2.605
**Maximum thickness (mm)**								
1.0	3.000	0.000	3.000	3.000	3.000	-	-	-
1.5	33.000	0.000	33.000	33.000	33.000	-	-	-
2.0	2.333	0.558	1.240	3.427	2.000	0.577	0.868	3.132
2.5	2.000	0.000	2.000	2.000	2.000	-	-	-
Overall	6.714	2.986	0.862	12.567	2.000	0.309	1.395	2.605

N—no-prep; P—prep.

## Data Availability

Additional data supporting the reported results can be requested from the corresponding authors.

## References

[1] Aslam A., Khan D.A., Hassan S.H., Ahmed B. (2017). Ceramic Fracture in Metal-Ceramic Restorations: The Aetiology. Dent. Update.

[2] Heffernan M.J., Aquilino S.A., Diaz-Arnold A.M., Haselton D.R., Stanford C.M., Vargas M.A. (2002). Relative translucency of six all-ceramic systems. Part I: Core materials. J. Prosthet. Dent..

[3] Edelhoff D., Sorensen J.A. (2002). Tooth structure removal associated with various preparation designs for anterior teeth. J. Prosthet. Dent..

[4] Silva L.H.D., Lima E., Miranda R.B.P., Favero S.S., Lohbauer U., Cesar P.F. (2017). Dental ceramics: A review of new materials and processing methods. Braz. Oral Res..

[5] Bosch G., Ender A., Mehl A. (2015). Non- and minimally invasive full-mouth rehabilitation of patients with loss of vertical dimension of occlusion using CAD/CAM: An innovative concept demonstrated with a case report. Int. J. Comput. Dent..

[6] Savi A., Turillazzi O., Crescini A., Manfredi M. (2014). Esthetic treatment of a diffuse amelogenesis imperfecta using pressed lithium disilicate and feldspathic ceramic restorations: 5-year follow up. J. Esthet. Restor. Dent..

[7] Tyas M.J., Anusavice K.J., Frencken J.E., Mount G.J. (2000). Minimal intervention dentistry—A review. FDI Commission Project 1-97. Int. Dent. J..

[8] Vailati F., Gruetter L., Belser U.C. (2013). Adhesively restored anterior maxillary dentitions affected by severe erosion: Up to 6-year results of a prospective clinical study. Eur. J. Esthet. Dent..

[9] McLaren E.A., Whiteman Y.Y. (2010). Ceramics: Rationale for material selection. Compend. Contin. Educ. Dent..

[10] McLaren E.A., LeSage B. (2011). Feldspathic veneers: What are their indications?. Compend. Contin. Educ. Dent..

[11] Layton D.M., Clarke M., Walton T.R. (2012). A systematic review and meta-analysis of the survival of feldspathic porcelain veneers over 5 and 10 years. Int. J. Prosthodont..

[12] Morimoto S., Albanesi R.B., Sesma N., Agra C.M., Braga M.M. (2016). Main Clinical Outcomes of Feldspathic Porcelain and Glass-Ceramic Laminate Veneers: A Systematic Review and Meta-Analysis of Survival and Complication Rates. Int. J. Prosthodont..

[13] Layton D.M., Walton T.R. (2007). The up to 21-year clinical outcome and survival of feldspathic porcelain veneers: Accounting for clustering. Int. J. Prosthodont..

[14] Peumans M., De Munck J., Fieuws S., Lambrechts P., Vanherle G., Van Meerbeek B. (2004). A prospective ten-year clinical trial of porcelain veneers. J. Adhes. Dent..

[15] Pini N.P., Aguiar F.H., Lima D.A., Lovadino J.R., Terada R.S., Pascotto R.C. (2012). Advances in dental veneers: Materials, applications, and techniques. Clin. Cosmet. Investig. Dent..

[16] Strassler H.E. (2007). Minimally invasive porcelain veneers: Indications for a conservative esthetic dentistry treatment modality. Gen. Dent..

[17] Smales R.J., Etemadi S. (2004). Long-term survival of porcelain laminate veneers using two preparation designs: A retrospective study. Int. J. Prosthodont..

[18] Wildgoose D.G., Winstanley R.B., van Noort R. (1997). The laboratory construction and teaching of ceramic veneers: A survey. J. Dent..

[19] Venturini A.B., Prochnow C., May L.G., Bottino M.C., Felipe Valandro L. (2015). Influence of hydrofluoric acid concentration on the flexural strength of a feldspathic ceramic. J. Mech. Behav. Biomed. Mater..

[20] Giordano R.A., Pelletier L., Campbell S., Pober R. (1995). Flexural strength of an infused ceramic, glass ceramic, and feldspathic porcelain. J. Prosthet. Dent..

[21] Gresnigt M., Ozcan M. (2011). Esthetic rehabilitation of anterior teeth with porcelain laminates and sectional veneers. J. Can. Dent. Assoc..

[22] Toreskog S. (2002). The minimally invasive and aesthetic bonded porcelain technique. Int. Dent. J..

[23] Touati B. (2005). Innovative dental ceramics: Expanding the material alternatives. Pract. Proced. Aesthet. Dent..

[24] Federizzi L., Gomes É.A., Báratro S.S., Baratto-Filho F., Bacchi A., Spazzin A.O. (2016). Use of Feldspathic Porcelain Veneers to Improve Smile Harmony: A 3-Year Follow-up Report. Braz. Dent. J..

[25] Giordano R. (2006). Materials for chairside CAD/CAM-produced restorations. J. Am. Dent. Assoc..

[26] Javaheri D. (2007). Considerations for planning esthetic treatment with veneers involving no or minimal preparation. J. Am. Dent. Assoc..

[27] Radz G.M. (2011). Minimum thickness anterior porcelain restorations. Dent. Clin. N. Am..

[28] Magne P., Belser U.C. (2004). Novel porcelain laminate preparation approach driven by a diagnostic mock-up. J. Esthet. Restor. Dent..

[29] Mozayek R.S., Alkhalil M.A., Allaf M., Dayoub S. (2019). Evaluation of the fracture strength of porcelain sectional veneers made from different sintered feldspathic porcelains: An in vitro study. Dent. Med. Probl..

[30] Farias-Neto A., Gomes E.M., Sánchez-Ayala A., Sánchez-Ayala A., Vilanova L.S. (2015). Esthetic Rehabilitation of the Smile with No-Prep Porcelain Laminates and Partial Veneers. Case Rep. Dent..

[31] Guess Gierthmuehlen P.C., Steger E. (2016). CAD/CAM Solutions for Minimally Invasive All-Ceramic Rehabilitation of Extended Erosive Lesions. Compend. Contin. Educ. Dent..

[32] da Cunha L.F., Gonzaga C.C., Saab R., Mushashe A.M., Correr G.M. (2015). Rehabilitation of the dominance of maxillary central incisors with refractory porcelain veneers requiring minimal tooth preparation. Quintessence Int..

[33] Agustín-Panadero R., Solá-Ruíz M.F., Chust C., Ferreiroa A. (2016). Fixed dental prostheses with vertical tooth preparations without finish lines: A report of two patients. J. Prosthet. Dent..

[34] Imburgia M., Canale A., Cortellini D., Maneschi M., Martucci C., Valenti M. (2016). Minimally invasive vertical preparation design for ceramic veneers. Int. J. Esthet. Dent..

[35] García-Gil I., Perez de la Calle C., Lopez-Suarez C., Pontevedra P., Suarez M.J. (2020). Comparative analysis of trueness between conventional and digital impression in dental-supported fixed dental prosthesis with vertical preparation. J. Clin. Exp. Dent..

[36] Vailati F., Belser U.C. (2008). Full-mouth adhesive rehabilitation of a severely eroded dentition: The three-step technique. Part 1. Eur. J. Esthet. Dent..

[37] Vailati F., Belser U.C. (2008). Full-mouth adhesive rehabilitation of a severely eroded dentition: The three-step technique. Part 2. Eur. J. Esthet. Dent..

[38] Vailati F., Belser U.C. (2008). Full-mouth adhesive rehabilitation of a severely eroded dentition: The three-step technique. Part 3. Eur. J. Esthet. Dent..

[39] Grütter L., Vailati F. (2013). Full-mouth adhesive rehabilitation in case of severe dental erosion, a minimally invasive approach following the 3-step technique. Eur. J. Esthet. Dent..

[40] D’Arcangelo C., Vadini M., D’Amario M., Chiavaroli Z., De Angelis F. (2018). Protocol for a new concept of no-prep ultrathin ceramic veneers. J. Esthet. Restor. Dent..

[41] Signore A., Kaitsas V., Tonoli A., Angiero F., Silvestrini-Biavati A., Benedicenti S. (2013). Sectional porcelain veneers for a maxillary midline diastema closure: A case report. Quintessence Int..

[42] Vadini M., D’Amario M., De Angelis F., Falco A., D’Arcangelo C. (2016). No-Prep Rehabilitation of Fractured Maxillary Incisors with Partial Veneers. J. Esthet. Restor. Dent..

[43] Piwowarczyk A., Blum J., Abendroth H. (2015). Non-prep restoration of an ankylosed incisor: A case report. Quintessence Int..

[44] Furuse A.Y., Soares J.V., Cunali R.S., Gonzaga C.C. (2016). Minimum intervention in restorative dentistry with V-shaped facial and palatal ceramic veneers: A clinical report. J. Prosthet. Dent..

[45] Ostermann F., Meyer G., Kern M. (2021). Survey of clinically used adhesive ceramic bonding methods—Follow up after 12 years. Dent. Mater..

[46] Sateia M.J. (2014). International classification of sleep disorders-third edition: Highlights and modifications. Chest.

[47] Gresnigt M.M.M., Cune M.S., Schuitemaker J., van der Made S.A.M., Meisberger E.W., Magne P., Özcan M. (2019). Performance of ceramic laminate veneers with immediate dentine sealing: An 11 year prospective clinical trial. Dent. Mater..

[48] Hickel R., Peschke A., Tyas M., Mjör I., Bayne S., Peters M., Hiller K.A., Randall R., Vanherle G., Heintze S.D. (2010). FDI World Dental Federation: Clinical criteria for the evaluation of direct and indirect restorations-update and clinical examples. Clin. Oral Investig..

[49] Cvar J.F., Ryge G. (2005). Reprint of criteria for the clinical evaluation of dental restorative materials. 1971. Clin. Oral Investig..

[50] Kasem A.T., Sakrana A.A., Ellayeh M., Özcan M. (2020). Evaluation of zirconia and zirconia-reinforced glass ceramic systems fabricated for minimal invasive preparations using a novel standardization method. J. Esthet. Restor. Dent..

[51] Abduo J. (2012). Safety of increasing vertical dimension of occlusion: A systematic review. Quintessence Int..

[52] Silverman M.M. (1951). Accurate measurement of vertical dimension by phonetics and the speaking centric space. Part I. Dent. Dig..

[53] Otto T., De Nisco S. (2002). Computer-aided direct ceramic restorations: A 10-year prospective clinical study of Cerec CAD/CAM inlays and onlays. Int. J. Prosthodont..

[54] Sjögren G., Molin M., van Dijken J.W. (2004). A 10-year prospective evaluation of CAD/CAM-manufactured (Cerec) ceramic inlays cemented with a chemically cured or dual-cured resin composite. Int. J. Prosthodont..

[55] Zimmer S., Göhlich O., Rüttermann S., Lang H., Raab W.H., Barthel C.R. (2008). Long-term survival of Cerec restorations: A 10-year study. Oper. Dent..

[56] Bindl A., Mörmann W.H. (2004). Survival rate of mono-ceramic and ceramic-core CAD/CAM-generated anterior crowns over 2-5 years. Eur. J. Oral Sci..

[57] Luciano M., Francesca Z., Michela S., Tommaso M., Massimo A. (2020). Lithium disilicate posterior overlays: Clinical and biomechanical features. Clin. Oral Investig..

[58] Lee B., Gadow R., Mitic V. (2017). Proceedings of the IV Advanced Ceramics and Applications Conference.

[59] Kürklü D., Azer S.S., Yilmaz B., Johnston W.M. (2013). Porcelain thickness and cement shade effects on the color and translucency of porcelain veneering materials. J. Dent..

[60] Igiel C., Weyhrauch M., Mayer B., Scheller H., Lehmann K.M. (2018). Effects of ceramic layer thickness, cement color, and abutment tooth color on color reproduction of feldspathic veneers. Int. J. Esthet. Dent..

[61] Sari T., Ural C., Yüzbasioglu E., Duran I., Cengiz S., Kavut I. (2018). Color match of a feldspathic ceramic CAD-CAM material for ultrathin laminate veneers as a function of substrate shade, restoration color, and thickness. J. Prosthet. Dent..

[62] Kim J.H., Park S.W., Lim H.P., Park C., Yun K.D. (2018). Biocompatibility Evaluation of Feldspathic Porcelain with Nano-Sized Silver Ion Particles. J. Nanosci. Nanotechnol..

[63] Sinescu C., Negrutiu M.L., Todea C., Balabuc C., Filip L., Rominu R., Bradu A., Hughes M., Podoleanu A.G. (2008). Quality assessment of dental treatments using en-face optical coherence tomography. J. Biomed. Opt..

[64] Ferrini F., Sannino G., Chiola C., Capparé P., Gastaldi G., Gherlone E.F. (2019). Influence of Intra-Oral Scanner (I.O.S.) on The Marginal Accuracy of CAD/CAM Single Crowns. Int. J. Environ. Res. Public Health.

